# Energy-Efficient Secure Communications for Wireless-Powered Cognitive Radio Networks

**DOI:** 10.3390/s21238040

**Published:** 2021-12-01

**Authors:** Kisong Lee

**Affiliations:** Department of Information and Communication Engineering, Dongguk University, Seoul 04620, Korea; kslee851105@gmail.com

**Keywords:** secrecy energy efficiency, energy harvesting, transmit power control, cognitive radio, low complexity

## Abstract

In this study, we investigate energy-efficient secure communications for wireless-powered cognitive ratio networks, in which multiple secondary users (SUs) share the same frequency band with primary users (PUs) and energy harvesting (EH) nodes harvest energy from the transmitted signals, even though information decoding is not permitted. To maximize the average secrecy energy efficiency (SEE) of SUs while ensuring acceptable interference on PUs and the required amount of energy for the EH nodes, we propose an energy-efficient transmit power control algorithm using dual decomposition, wherein suboptimal transmit powers are determined in an iterative manner with low complexity. Through extensive simulations in various scenarios, we verify that the proposed scheme has a higher average SEE than conventional schemes and a considerably shorter computation time than the optimal scheme.

## 1. Introduction

Cognitive radio networks (CRNs) have emerged as an efficient tool for improving spectrum utilization through enablement of opportunistic access to vacant licensed spectrum bands [1]. In CRNs, unlicensed secondary users (SUs) can share the spectrum with primary users (PUs) as long as they do not interfere with the transmission of the PUs. In other words, it is important for SUs to maintain their interference on PUs below a permissible level. Accordingly, various strategies for spectrum sharing have been investigated, such as cooperative spectrum sensing [2], dynamic spectrum access [3], and interference mitigation [4,5].

The advancement of 5G communication systems has resulted in emphasis being placed on the importance of energy efficiency (EE) because energy consumption increases with an increase in the various functions of mobile devices. Several studies have been conducted to improve the EE of CRNs [6,7,8,9]. In particular, the spectral-energy efficiency tradeoff for CRNs was analyzed in [6], and energy-efficient transmit power control (TPC) for orthogonal frequency-division multiplexing (OFDM)-based CRNs was proposed in [7,8]. Resource allocation was studied in [9] to maximize the EE of dynamic access for intra-cluster and inter-cluster data transmissions.

With the development of the Internet of Things (IoT), it is predicted that more than tens of billions of wireless devices will be connected through the Internet to collect and share information to provide convenience and services to users [10]. However, it can be inconvenient for users as the batteries of many devices need to be recharged or replaced frequently in these IoT systems. Therefore, in addition to the efficient utilization of communication resources, energy harvesting (EH) technology has attracted great attention as a means of providing sustainable energy to wireless devices [11]. Accordingly, several attempts have been made to improve the EE of wireless-powered CRNs (WPCRNs), in which the nodes are capable of EH [12,13,14,15]. In [12], cooperative scheduling and power control were designed to maximize the EE of the SU’s uplink transmission. In [13,14], EE optimization problems for non-orthogonal multiple access (NOMA)-based WPCRNs were developed and energy-efficient resource allocations were proposed. In [15], a joint optimization of spectrum sensing and transmit power was investigated to maximize the EE of EH-enabled SUs.

Spectrum sharing between different networks also increases the risk of eavesdropping; therefore, some studies have examined physical layer security to ensure information confidentiality without reliance on the secret key in CRNs [16,17,18,19,20]. In [16], the security–reliability tradeoff of multiuser scheduling in WPCRNs was analyzed under consideration of channel-aware user scheduling and energy-aware user scheduling. In [17], the transmit beamforming vector and power splitting ratio were optimized to maximize the outage-constrained secrecy rate (SR) under the constraints of EH and interference on PUs. In [18], a cooperative jammer-aided transmission scheme was proposed to enhance the secrecy performance of cooperative WPCRNs. In [19,20], secrecy EE (SEE) maximization problems were solved by optimization techniques. However, the existing strategies [17,18,19,20] for secure communications for CRNs have been proposed on the basis of a centralized approach, because of which their implementation introduces enormous computational complexity. Although low complexity TPC strategies were also investigated in [21,22], these schemes achieved only sub-optimal performances slightly away from the optimal. Therefore, realization of practical WPCRNs necessitates development of a security-aware energy-efficient TPC algorithm that can be implemented with low complexity.

In this study, we investigate energy-efficient secure communications for WPCRNs, in which multiple SUs share the same frequency band with PUs and EH nodes are permitted to only harvest energy from the signals transmitted by transmitters (Txs). Considering that the EH nodes are untrusted nodes that are not permitted to decode secret information, we formulate the problem for deriving the optimal TPC strategy for SU Txs in order to maximize the average SEE of SUs while ensuring permissible interference on the PU receiver (Rx) and the minimum amount of energy for the EH nodes. To overcome the non-convexity of the problem caused by the fractional objective function and interference term, we use dual decomposition and nonlinear fractional programming for deriving the suboptimal transmit power in analytical form. Based on the derived result, we propose an energy-efficient TPC algorithm that can be implemented in an iterative manner with low complexity. Results of extensive simulations confirm that the proposed scheme shows performance closest to the optimal scheme, and that it outperforms the conventional scheme while having a significantly shorter computation time than the optimal scheme.

The remainder of this paper is organized as follows. In Section 2, we present the SEE maximization problem along with the system model of WPCRNs. In Section 3, we propose the energy-efficient TPC algorithm for secure communications for WPCRNs. In Section 4, we present performance evaluations performed on the basis of extensive simulations, and make our conclusions in Section 5.

## 2. System Model and Problem Statement

**Figure 1:**
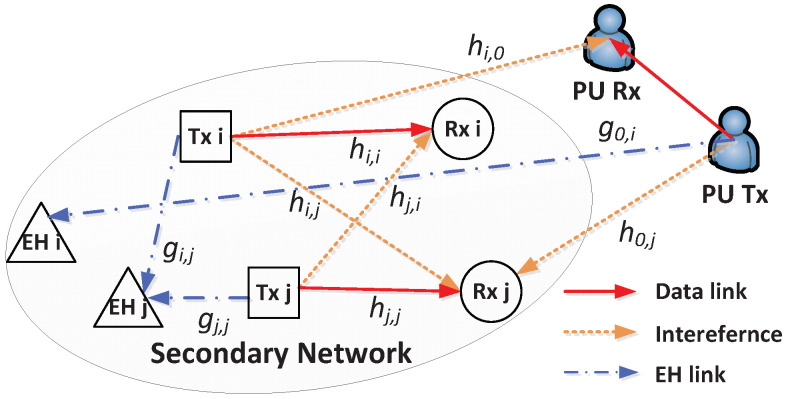
System model of WPCRNs for secure communications with N=2.

As shown in Figure 1, multiple SU pairs share the same frequency band with PUs while ensuring that the interference on the PUs remains below a predetermined threshold. At the same time, EH nodes are licensed to harvest energy from signals sent by Txs but not to interpret information. In other words, sufficient energy must be guaranteed for the sustainable operation of the EH nodes, but they are untrusted nodes from the viewpoint of information transfer [23,24]. If each EH node acts as an eavesdropper and attempts to decode confidential information shared between a pair of SUs, the secure communication cannot be guaranteed. In order to prevent this eavesdropping from occurring, it is necessary to optimize the transmit powers of the SU Txs so as to ensure the confidentiality of information against the EH nodes while providing sufficient energy to them.

All nodes are equipped with a single antenna, and the set of SU pairs is denoted as N, i.e., |N|=N. Moreover, the EH nodes are associated with each SU pair. The channel gain between SU Tx *i* and SU Rx *j* is denoted as $h_{i,j}$ and that between SU Tx *i* and EH node *j* is denoted as $g_{i,j}$. Moreover, the index 0 is assigned for PUs; that is, $h_{i,0}$ is the channel gain between SU Tx *i* and the PU Rx whereas $g_{0,j}$ is the channel gain between the PU Tx and EH node *j*. We assume that each channel follows a discrete time block-fading model and that $h_{i,0}$ is known at SU Tx *i* so as to maintain the interference on the PU Rx below the permissible level [25]. The additive white Gaussian noise at each receiving node is also modeled as $z∼\mathrm{CN}\;\left(0,\sigma^{2}\right)$.

From the Shannon capacity, the achievable spectral efficiency (SE) of SU pair *i* is obtained as

(1) $$\begin{array}{cc} r_{i} & =\log_{2}\left(1+\frac{p_{i}{\left|h_{i,i}\right|}^{2}}{\sum_{k\in N\setminus \{i\}}p_{k}|h_{k,i}{|}^{2}+p_{0}{\left|h_{0,i}\right|}^{2}+\sigma^{2}}\right), \end{array}$$

where $p_{i}$ and $p_{0}$ are the transmit powers of SU Tx *i* and the PU Tx, respectively.

The interference caused by SU Tx *i* on the PU Rx is expressed as

(2) $$\begin{array}{cc} I_{i} & =p_{i}{\left|h_{i,0}\right|}^{2}. \end{array}$$

Each EH node can harvest energy from the signals transmitted by the PU Tx as well as the SU Txs; therefore, the total amount of energy collected from EH node *i* is expressed as

(3) $$\begin{array}{cc} Q_{i} & =\zeta_{i}\left(p_{0}|g_{0,i}{|}^{2}+\sum_{j\in N}p_{j}{\left|g_{j,i}\right|}^{2}\right), \end{array}$$

where $\zeta_{i}$ is the energy conversion efficiency of EH node *i*. However, if EH node *i* attempts to eavesdrop on information contained in the signal transmitted by SU Tx *i* instead of harvesting energy, the achievable SE is given by

(4) $$\begin{array}{cc} r_{i}^{e} & =\log_{2}\left(1+\frac{p_{i}{\left|g_{i,i}\right|}^{2}}{\sum_{k\in N\setminus \{i\}}p_{k}|g_{k,i}{|}^{2}+p_{0}{\left|g_{0,i}\right|}^{2}+\sigma^{2}}\right). \end{array}$$

Using (1) and (4), we can define the SR of SU pair *i* as the difference between $r_{i}$ and $r_{i}^{e}$ [26], which can be formulated as

(5) $$\begin{array}{cc} r_{i}^{s} & ={\left[r_{i}-r_{i}^{e}\right]}^{+}, \end{array}$$

where ${\left[\cdot \right]}^{+}=\max\left(0,\cdot \right)$.

Furthermore, the total power consumed by SU pair *i* can be expressed as

(6) $$\begin{array}{cc} p_{i}^{\mathrm{CE}} & =p_{C}+p_{i}, \end{array}$$

where $p_{C}$ is the constant energy consumed by the circuits of each SU pair.

Using (5) and (6), we define the SEE of SU pair *i* as the ratio of the SR to the total power consumption (bits/Hz/Joule).

(7) $$\begin{array}{cc} \eta_{i}^{s} & =\frac{r_{i}^{s}}{p_{i}^{\mathrm{CE}}}. \end{array}$$

The definition of the SEE is used to evaluate how efficiently energy can be utilized to transmit confidential information.

To ensure information confidentiality against the untrusted EH nodes and simultaneously improve the EE of the SU pairs, we develop the following problem that determines the optimal transmit powers of the SU Txs for maximizing the average SEE under the constraints of permissible interference on PUs, $I_{\mathrm{th}}$, and the minimum amount of energy for each EH node, $Q_{\min}$:

(8) $$\begin{array}{ccc} \max_{0⪯\vec{p}} & \; & \frac{1}{N}\sum_{i\in N}\eta_{i}^{s}\\ s.t. & \; & \sum_{i\in N}I_{i}\leq I_{\mathrm{th}}\\ \;\;\; & \; & Q_{i}\geq Q_{\min},\;\;i\in N\\ \;\;\; & \; & p_{i}\leq P_{\max},\;\;i\in N, \end{array}$$

where $\vec{p}=\left\{p_{1},p_{2},\cdots ,p_{N}\right\}$ and $P_{\max}$ is the maximum transmit power of each SU Tx. However, it is difficult to mathematically derive the optimal solution of $\vec{p}$ because the problem (8) is non-convex owing to the fractional objective function and co-channel interference.

## 3. Energy-Efficient Transmit Power Control Algorithm

In this section, we propose an energy-efficient TPC algorithm that can determine suboptimal transmit powers with low complexity.

We first decompose the original problem (8) into *N* subproblems, and then solve each subproblem independently [27]. In the subproblem, the objective is to maximize the SEE of individual SU pair and $I_{\mathrm{th}}$ in the first constraint of (8) is changed to $\frac{I_{\mathrm{th}}}{N}$, such that the constraint of permissible interference on PUs can be satisfied even when each subproblem is solved independently.

(9) $$\begin{array}{ccc} \max_{0\leq p_{i}} & \; & \eta_{i}^{s}\\ s.t. & \; & C1:\;I_{i}\leq \frac{I_{\mathrm{th}}}{N}\\ \;\;\; & \; & C2:\;Q_{i}\geq Q_{\min}\\ \;\;\; & \; & C3:\;p_{i}\leq P_{\max}. \end{array}$$

By defining $x_{i}=\frac{r_{i}^{s}}{p_{i}^{\mathrm{CE}}}$ and using nonlinear fractional programming [28], we can translate the objective function of (9) from a fractional form to an equivalent subtractive form, $r_{i}^{s}-x_{i}p_{i}^{\mathrm{CE}}$. Then, we can reformulate the subproblem (9) as

(10) $$\begin{array}{ccc} \max_{0\leq p_{i}} & \; & r_{i}^{s}-x_{i}p_{i}^{\mathrm{CE}}\\ s.t. & \; & C1,\;C2,\;\mathrm{and}\;C3. \end{array}$$

To derive the transmit power of each SU Tx from (10), we define the Lagrangian function of (10) as follows:

(11) $$\begin{array}{cc} L(p_{i},\lambda_{i},\mu_{i},\kappa_{i})\; & =\;r_{i}^{s}\;-\;x_{i}p_{i}^{\mathrm{CE}}\;+\;\lambda_{i}\;\left(\frac{I_{\mathrm{th}}}{N}\;-\;I_{i}\right)+\mu_{i}\;\left(Q_{i}\;-\;Q_{\min}\right)+\kappa_{i}\;\left(P_{\max}\;-\;p_{i}\right), \end{array}$$

where $\lambda_{i}\geq 0$, $\mu_{i}\geq 0$, and $\kappa_{i}\geq 0$ are, respectively, the Lagrange multipliers of each constraint of the subproblem (10).

In addition, we formulate the dual problem as

(12) $$\begin{array}{c} \min_{0\leq \lambda_{i},\;0\leq \mu_{i},\;0\leq \kappa_{i}}\;\;G\left(\lambda_{i},\mu_{i},\kappa_{i}\right), \end{array}$$

where $G\left(\lambda_{i},\mu_{i},\kappa_{i}\right)=\max_{0\leq p_{i}}\;\;L\left(p_{i},\lambda_{i},\mu_{i},\kappa_{i}\right)$. Determination of the suboptimal transmit power of the SU Tx necessitates updating of $p_{i}$ to maximize $L(p_{i},\lambda_{i},\mu_{i},\kappa_{i})$ and updating of $\lambda_{i}$, $\mu_{i}$, and $\kappa_{i}$ to minimize $G(\lambda_{i},\mu_{i},\kappa_{i})$ in an iterative manner.

By taking the derivative of (11) with respect to $p_{i}$, we can derive the transmit power that satisfies the Karush–Kuhn–Tucker (KKT) conditions as follows:

(13) $$\begin{array}{c} p_{i}\;=\;{\left[\frac{1}{\ln2\;\left(x_{i}(1\;-\;\zeta_{i}|g_{i,i}{|}^{2})\;+\;\lambda_{i}|h_{i,0}{|}^{2}\;-\;\mu_{i}\zeta_{i}{\left|g_{i,i}\right|}^{2}\;+\;\kappa_{i}\right)\;+\;t_{i}^{\left[s\right]}}\;-\;\frac{\Omega_{i}}{|h_{i,i}{|}^{2}}\right]}^{+}, \end{array}$$

where $\Omega_{i}=\sigma^{2}+p_{0}|h_{0,i}{|}^{2}+\sum_{j\in N\setminus \{i\}}p_{j}{\left|h_{j,i}\right|}^{2}$ and $t_{i}^{\left[s\right]}$ is defined as

(14) $$\begin{array}{c} t_{i}^{\left[s\right]}=\frac{|g_{i,i}{|}^{2}}{\Psi_{i}}, \end{array}$$

where $\Psi_{i}=\sigma^{2}+p_{0}|g_{0,i}{|}^{2}+\sum_{l\in N}p_{l}{\left|g_{l,i}\right|}^{2}$. Given that $\Omega_{i}$ is the sum of interference and noise powers at SU Rx *i* and $\Psi_{i}$ is the total received power at EH node *i*, $p_{i}$ can be easily determined as in (13) through sharing of information between SU pair *i* and its associated EH node without other SU pairs j∈N∖{i}. This enables the proposed algorithm to calculate the suboptimal transmit power with low complexity.

A gradient algorithm can also be used to update the Lagrange multipliers as follows:

(15) $$\begin{array}{cc} \; & \lambda_{i}\leftarrow {\left[\lambda_{i}-\alpha_{1}\left(\frac{I_{\mathrm{th}}}{N}-I_{i}\right)\right]}^{+},\\ \; & \mu_{i}\leftarrow {\left[\mu_{i}-\alpha_{2}\left(Q_{i}-Q_{\min}\right)\right]}^{+},\\ \; & \kappa_{i}\leftarrow {\left[\kappa_{i}-\alpha_{3}\left(P_{\max}-p_{i}\right)\right]}^{+}, \end{array}$$

where $\alpha_{1}$, $\alpha_{2}$, and $\alpha_{3}$ are step sizes that are sufficiently small for an appropriate update.

The proposed algorithm operates as described in Algorithm 1 to determine the suboptimal transmit powers of the SU Txs. The computational complexity of this algorithm can be analyzed as follows. In the worst case, $\epsilon^{-2}$ iterations are needed to make the norm of the gradient smaller than ϵ [29]; therefore, $\epsilon^{-2}$ iterations are required for the convergence of the inner loop (from line 4 to line 11). *T* is defined as the number of iterations needed for the convergence of the outer loop (from line 2 to line 13) [30]. Then, the computational complexity of the proposed algorithm is given as $O\;\left(TN\epsilon^{-2}\right)$, where *N* is the number of calculations required to compute $\vec{p}$.
**Algorithm 1** Proposed energy-efficient transmit power control algorithm.1:  Initialize $\vec{p}$, $\vec{\lambda }$, $\vec{\mu }$, and $\vec{\kappa }$ randomly2:  **repeat**3:     Set $\vec{x}={\vec{r}}^{s}/{\vec{p}}^{\mathrm{CE}}$4:     **repeat**5:        ${\vec{p}}_{\mathrm{old}}\leftarrow \vec{p}$6:        **for** i=1 to *N*7:           Compute $p_{i}$ according to (13)8:           Update $\lambda_{i}$, $\mu_{i}$, and $\kappa_{i}$ according to (15)9:        **end for**10:       $\vec{p}=\left\{p_{1},p_{2},\cdots ,p_{N}\right\}$11:    **until** $∥\vec{p}-{\vec{p}}_{\mathrm{old}}∥<\epsilon$12:    Update ${\vec{r}}^{s}$ and ${\vec{p}}^{\mathrm{CE}}$ with $\vec{p}$13: **until** $∥{\vec{r}}^{s}-\vec{x}{\vec{p}}^{\mathrm{CE}}∥<\delta$

## 4. Simulation Results and Discussion

In our simulations, the following system parameters are used as default unless stated otherwise: *N* = 3, $P_{\max}$ = $p_{0}=P_{C}$ = 30 dBm, $\sigma^{2}=-100$ dBm, $E_{\min}=-15$ dBm, $I_{\max}=-50$ dBm, and $\zeta_{i}$ = 0.5 for i∈N [11,15,25]. We distribute the SUs and EH nodes randomly over an area of 50 × 50 m, and the maximum distances of signal link in the same pair and the associated EH link are set to 15 and 10 m, respectively. Moreover, the secondary network is on average 1 km away from PUs for effective spectrum sharing. A scenario of urban areas is assumed, and accordingly, a simplified path loss model with a path loss exponent of 2.7 is considered [23]. In addition, Rayleigh fading is used for the signal links whereas Rician fading with a *K*-factor of 6 is used for the EH links to reflect multi-path fading [11]. The average SEE is also used as a performance metric, which can be mathematically written as $E\left[\left({𝟙}_{\sum_{i\in N}I_{i}\leq I_{\mathrm{th}}}\right)\cdot \left(\prod_{i\in N}{𝟙}_{Q_{i}\geq Q_{\min}}\right)\cdot \frac{1}{N}\sum_{i\in N}\eta_{i}^{s}\right]$. The average SEE is set to zero if the constraint of $I_{\mathrm{th}}$ or $Q_{\min}$ is violated; thus, the impact of violation of both the constraints is inherent in the calculation of the average SEE. The following five schemes are compared for performance evaluation.

- Optimal scheme: under the assumption of perfect channel state information (CSI), the near-optimal performance can be determined by brute-force search, in which all combinations are evaluated by quantizing $\vec{p}$ into M=100 equally spaced values. Therefore, the computational complexity of this scheme increases exponentially with *N*, i.e., $O\;\left(M^{N}\right)$.
- Proposed scheme: the transmit powers of SU Txs are determined using Algorithm 1.
- SR max. scheme: the transmit powers of SU Txs are determined to maximize the average SR, $\frac{1}{N}\sum_{i\in N}r_{i}^{s}$, which is determined by brute-force search.
- On–off scheme [21]: the transmit powers of SU Txs are determined to be either $P_{\max}$ or 0 so as to maximize the average SEE.
- Equally reduced power (ERP) scheme [22]: all SU Txs use the same transmit power that maximizes the average SEE while satisfying all constraints, and the optimal value of the transmit power is determined by one-dimensional brute-force search.
- Max. power scheme: the transmit powers of SU Txs are determined as $P_{\max}$.

**Figure 2:**
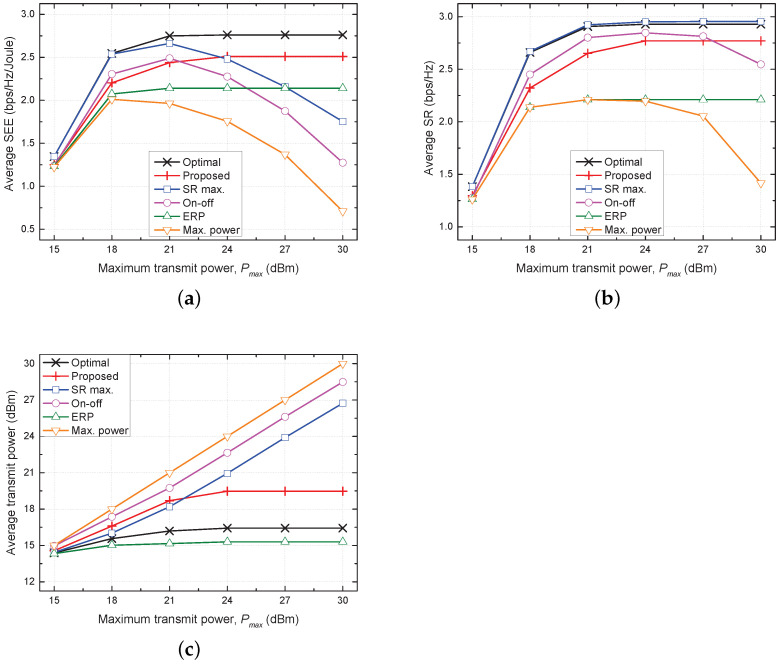
Comparison of performances of five considered schemes as a function of maximum transmit power ($P_{\max}$). (**a**) Average SEE vs. $P_{\max}$. (**b**) Average SR vs. $P_{\max}$. (**c**) Average transmit power vs. $P_{\max}$.

Figure 2 shows plots of the average SEE, average SR, and average transmit power versus the maximum transmit power ($P_{\max}$) for all five evaluated schemes. In the optimal, proposed, and ERP schemes, the average SEE converges to respective stationary points even when $P_{\max}$ increases by more than 24 dBm. This result indicates that it is not beneficial to increase the transmit power for improving the SEE, because the growth rate of energy consumption is much larger than that of the average SR when $P_{\max}\geq 24$ dBm. Because of the efficient use of transmit power, the proposed scheme shows performance closest to the optimal scheme and the ERP scheme shows the best performance among the conventional schemes. On the other hand, the SR max. scheme uses more transmit power to maximize the average SR as $P_{\max}$ increases, but the increase in the average SR is limited by severe interference. For a similar reason, in the on–off and max. power schemes, the average SR decreases even when they use more transmit power because these schemes do not perform adaptive TPC. Consequently, because of inefficient use of energy in these conventional schemes, the average SEE degrades rapidly with increasing $P_{\max}$.

**Figure 3:**
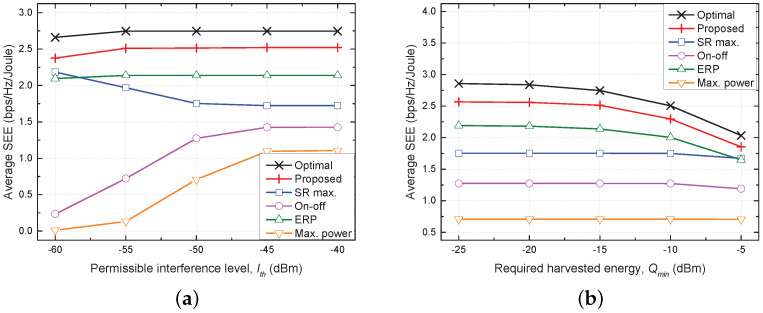
Comparison of performances of five considered schemes as a function of constraints of $I_{\mathrm{th}}$ and $Q_{\min}$. (**a**) Average SEE vs. $I_{\mathrm{th}}$. (**b**) Average SEE vs. $Q_{\min}$.

Figure 3 shows plots of the average SEE versus the permissible interference level ($I_{\mathrm{th}}$) and the required harvested energy ($Q_{\min}$) for all five evaluated schemes. In environments in which satisfaction of these constraints is more difficult, e.g., lower $I_{\mathrm{th}}$ and larger $Q_{\min}$, the constraints are frequently not satisfied, and this violation imposes a penalty of the average SEE being set to 0. Therefore, the average SEE of most of the considered schemes decreases as $I_{\mathrm{th}}$ decreases or $Q_{\min}$ increases. However, we also observe that the average SEE of the SR max. scheme increases even when $I_{\mathrm{th}}$ decreases. The SR max. scheme should inevitably reduce the transmit power with decreasing $I_{\mathrm{th}}$ in order to decrease the amount of interference on the PUs. This reduction in the transmit power is detrimental to the SR, but it improves the SEE. The fact that the proposed scheme shows performance closest to the optimal scheme over the complete ranges of $I_{\mathrm{th}}$ and $Q_{\min}$ confirms the effectiveness of the proposed TPC strategy.

**Figure 4:**
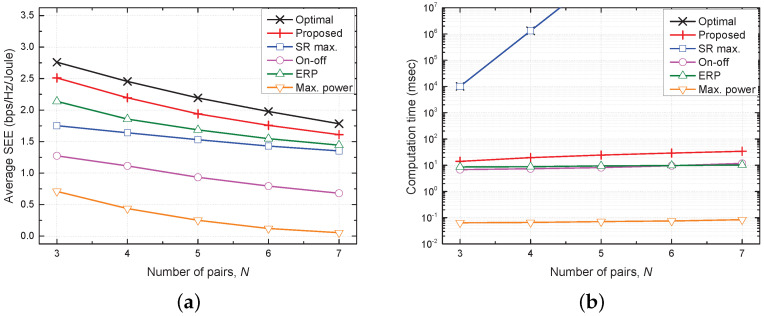
Comparison of performances of five considered schemes as a function of number of SU pairs (*N*). (**a**) Average SEE vs. *N*. (**b**) Computation time vs. *N*.

Figure 4 shows plots of the average SEE and computation time versus the number of SU pairs (*N*). As *N* increases, the concurrent transmissions among SU pairs cause severe mutual interference. This limits the improvement in the average SR relative to the transmit power used, and therefore, the average SEE decreases with increasing *N*. The max. power scheme has the shortest computation time because it uses a fixed transmit power, but its SEE is also lowest because of inefficient TPC. The on–off and ERP schemes have slightly shorter computation times than the proposed scheme, but their performances are much lower than that of the proposed scheme. The performance of the proposed scheme is about 10% lower than that of the optimal scheme because of the distributed nature of the former; however, its performance is still closest to that of the optimal scheme because it performs effective TPC with a significantly shorter computation time. This result validates the effectiveness of the proposed scheme with respect to the SEE and computational complexity.

## 5. Conclusions

In this study, we focused on the enhancement of both the EE and secure communications for WPCRNs. To this end, we formulated a TPC problem that maximizes the average SEE while ensuring acceptable interference on PUs and the required amount of energy for the EH nodes. We derived the suboptimal transmit powers analytically using dual decomposition, and proposed an energy-efficient TPC algorithm with low complexity. Simulation results demonstrated that the proposed scheme, by virtue of performing TPC in consideration of the SR and EE, can achieve higher average SEE than the conventional schemes, e.g., more than 10% in default settings. Furthermore, the computation time of the proposed scheme is considerably shorter than that of the optimal scheme, which is less than tens of milliseconds even if the number of nodes is large. We expect our solution to be of use in solving energy shortage and information security in practical IoT systems.

## Data Availability

Not applicable.

## References

[1] Haykin S. (2005). Cognitive radio: Brain-empowered wireless communications. IEEE J. Sel. Commun..

[2] Xie S., Liu Y., Zhang Y., Yu R. (2010). A parallel cooperative spectrum sensing in cognitive radio networks. IEEE Trans. Veh. Technol..

[3] Ji Z., Liu K.J.R. (2007). Dynamic spectrum sharing: A game theoretical overview. IEEE Commun. Mag..

[4] Cheng S., Ao W.C., Chen K. (2011). Efficiency of a cognitive radio link with opportunistic interference mitigation. IEEE Trans. Wirel. Commun..

[5] Kpojime H.O., Safdar G.A. (2015). Interference mitigation in cognitive-radio-based femtocells. IEEE Commun. Surv. Tutor..

[6] Haider F., Wang C., Haas H., Hepsaydir E., Ge X., Yuan D. (2015). Spectral and energy efficiency analysis for cognitive radio networks. IEEE Trans. Wirel. Commun..

[7] Wang Y., Xu W., Yang K., Lin J. (2012). Optimal energy-efficient power allocation for OFDM-based cognitive radio networks. IEEE Commun. Lett..

[8] Mao J., Xie G., Gao J., Liu Y. (2013). Energy efficiency optimization for OFDM-based cognitive radio systems: A water-filling factor aided search method. IEEE Trans. Wirel. Commun..

[9] Ren J., Zhang Y., Zhang N., Zhang D., Shen X. (2016). Dynamic channel access to improve energy efficiency in cognitive radio sensor networks. IEEE Trans. Wirel. Commun..

[10] Chen X., Ng D.W.K., Yu W., Larsson E.G., Al-Dhahir N., Schober R. (2021). Massive access for 5G and beyond. IEEE J. Sel. Areas Commun..

[11] Lu X., Wang P., Niyato D., Kim D.I., Han Z. (2015). Wirel. networks with RF energy harvesting: A contemporary survey. IEEE Commun. Surv. Tutor..

[12] Yin S., Qu Z., Wang Z., Li L. (2017). Energy-efficient cooperation in cognitive Wirel. powered networks. IEEE Commun. Lett..

[13] Zhao W., She R., Bao H. (2019). Energy efficiency maximization for two-way relay assisted CR-NOMA system based on SWIPT. IEEE Access.

[14] Wang X., Na Z., Lam K.Y., Liu X., Gao Z., Li F., Wang L. (2019). Energy efficiency optimization for NOMA-based cognitive radio with energy harvesting. IEEE Access.

[15] Lee K., Yoon C., Jo O., Lee W. (2018). Joint optimization of spectrum sensing and transmit power in energy harvesting-based cognitive radio networks. IEEE Access.

[16] Ding X., Zou Y., Zhang G., Chen X., Wang X., Hanzo L. (2019). The security–reliability tradeoff of multiuser scheduling-aided energy harvesting cognitive radio networks. IEEE Trans. Commun..

[17] Ni L., Da X., Hu H., Huang Y., Xu R., Zhang M. (2018). Outage constrained robust transmit design for secure cognitive radio with practical energy harvesting. IEEE Access.

[18] Chen X., Guo L., Li X., Dong C., Lin J., Mathiopoulos P.T. (2018). Secrecy rate optimization for cooperative cognitive radio networks aided by a Wirel. energy harvesting jammer. IEEE Access.

[19] Ouyang J., Lin M., Zou Y., Zhu W., Massicotte D. (2017). Secrecy energy efficiency maximization in cognitive radio networks. IEEE Access.

[20] Ni L., Da X., Hu H., Zhang M., Cumanan K. (2020). Outage constrained robust secrecy energy efficiency maximization for EH cognitive radio networks. IEEE Wirel. Commun. Lett..

[21] Gjendemsjø A., Gesbert D., Øien G.E., Kiani S.G. (2008). Binary power control for sum rate maximization over multiple interfering links. IEEE Trans. Wirel. Commun..

[22] Lee W., Ban T., Jung B.C. (2019). Distributed transmit power optimization for device-to-device communications underlying cellular networks. IEEE Access.

[23] Kalamkar S.S., Banerjee A. (2017). Secure communication via a Wirel. energy harvesting untrusted relay. IEEE Trans. Veh. Technol..

[24] Lee K., Hong J.P., Lee W. (2021). Deep learning framework for secure communication with an energy harvesting receiver. IEEE Trans. Veh. Technol..

[25] Lee W., Lee K. (2021). Resource allocation scheme for guarantee of QoS in D2D communications using deep neural network. IEEE Commun. Lett..

[26] Wyner A.D. (1975). The wire-tap channel. Bell Syst. Tech. J..

[27] Yu W., Lui R. (2006). Dual methods for nonconvex spectrum optimization of multicarrier systems. IEEE Trans. Commun..

[28] Dinkelbach W. (1967). On nonlinear fractional programming. Manag. Sci..

[29] Fliege J., Vaz A.I.F., Vicente L.N. (2018). Complexity of gradient descent for multiobjective optimization. Optim. Methods Softw..

[30] Venturino L., Prasad N., Wang X. (2009). Coordinated scheduling and power allocation in downlink multicell OFDMA networks. IEEE Trans. Veh. Technol..

